# Intravitreal Antiangiogenic Treatment for Diabetic Retinopathy: A Mexican Real-Life Scenario Experience

**DOI:** 10.3390/life14080976

**Published:** 2024-08-02

**Authors:** Sonia López-Letayf, Oscar Vivanco-Rojas, Valentina Londoño-Angarita, Fátima Sofía Magaña-Guerrero, Beatriz Buentello-Volante, Yonathan Garfias

**Affiliations:** 1 Department of Biochemistry, Faculty of Medicine, Universidad Nacional Autónoma de México, Av. Universidad 3000, Mexico City 04510, Mexico; sonyletayf@comunidad.unam.mx (S.L.-L.); oscarv@bq.unam.mx (O.V.-R.); 2 Cell and Tissue Biology, Research Unit, Institute of Ophthalmology, Conde de Valenciana, Chimalpopoca 14, Mexico City 06800, Mexico; dra.vlondono@gmail.com (V.L.-A.); fatima.magana@institutodeoftalmologia.org (F.S.M.-G.); bbuentello@institutodeoftalmologia.org (B.B.-V.)

**Keywords:** diabetic retinopathy, anti-VEGF, ranibizumab, aflibercept, diabetic macular edema, Mexican population

## Abstract

The objective of this study was to analyze the effectiveness of two intravitreal antiangiogenic drugs, ranibizumab and aflibercept, in a Mexican population over a period of 5 years, evaluating the improvement in visual acuity (VA) and central retinal thickness (CRT) in a real-world scenario. This is a retrospective study with subjects diagnosed with diabetic retinopathy (DR), proliferative diabetic retinopathy (PDR), and diabetic macular edema (DME) receiving intravitreal injections of ranibizumab and/or aflibercept. In this study, we analyzed 588 eyes of 294 patients who received intravitreal antiangiogenic injections. The results showed an improvement regardless of antiangiogenic treatment or diagnosis in both VA and CRT. We found that both aflibercept and ranibizumab improved VA, while subjects with DME responded less to antiangiogenic treatment (*p* < 0.05), and that this difference did not correspond to the CRT measured by OCT. These results support evidence that intravitreal antiangiogenic medications are effective for ophthalmic complications of diabetes in our population; however, damage to visual structures is not reversed in most patients. And that the perception by the patient (VA) and that of the ophthalmologist (CRT) do not completely correlate in our study.

## 1. Introduction

Diabetes mellitus is a disease characterized by chronically high blood glucose levels, with a global prevalence of 10.5% until 2021, with an estimated 12.2% of the world’s population being affected by diabetes mellitus by 2048. Mexico is among the first 10 countries with a high rate of diabetes [1], with a prevalence of 18.3% [2]. A hyperglycemic state causes microvascular changes, causing complications such as neuropathy, retinopathy (DR), and diabetic nephropathy (DN) [3].

DR is a microvascular complication, with a prevalence in North America and the Caribbean of 33.3% in diabetics; this prevalence is projected to increase by 2030 [4,5]. Neural, inflammatory, and vascular dysfunctions of retinal tissue are the main causes of DR pathogenesis. The latter are associated with retinal blood vessel permeability, with vascular endothelial growth factor (VEGF) being one of the major molecules involved in this endothelial permeability.

This factor is responsible for maintaining endothelial cell survival, proliferation, and migration, as well as the growth of the newly formed vessels. It serves as the main therapeutic target for the treatment of this disease; however, it can only be used in advanced stages of DR and in diabetic macular edema (DME) [6]. Currently, the only means of preventing the early stages from advancing to a more severe stage is systemic risk factor control [5]. DME is a complication that can appear at any stage of DR, and it is defined as a thickening of the retina that involves the fovea due to abnormal liquid accumulation. It is the main reason for vision loss among patients with DR, with a prevalence of 5.47% globally among people with diabetes [7].

The VEGF-A isoform has the greatest angiogenic effect and is the main therapeutic target for the treatment of DR and DME. VEGF dysregulation is dependent on multiple factors, but damage also involves neuronal damage and injury to immune components [6]. Ranibizumab (Lucentis, Novartis Pharma Stein AG, Switzerland) is a humanized recombinant monoclonal antibody, and aflibercept (Wetlia, Bayer AG, Germany) is a recombinant fusion protein. The latter not only recognizes VEGF-A but also targets placental growth factor (PGF), which has been associated with the progression of DR [8]. While anti-VEGF biologicals are used to treat neovascularization, they also inhibit blood vessel permeability, which is another consequence of the vascular changes that are present in DR. DME is a complication of increased permeability, DR, which increases the risk of at any point presenting DME, and at the same time, having DME increases the risk of hypoxic events that lead to the progression of DR. Therefore, these two entities are mutually engaged. Pan-retinal photocoagulation (PRP) is usually used in conjunction with intravitreal anti-VEGF biologicals. How PRP works is not fully understood, but it is believed that the laser causes some cells to die in areas of high oxygen demand, diminishing hypoxia signals in areas of poor retinal perfusion. This exerts a decrease in signals that would lead to neovascularization, such as VEGF [9].

Although the use of intravitreal anti-VEGF biologicals to treat DM complications such as DR and DME has been accepted in the medical community, there are still controversies about their effectiveness. For example, Antoszyk, M., and colleagues showed that intravitreal aflibercept was equally effective as PRP in proliferative DR with vitreous hemorrhage in a 24-week follow-up [10]. Moreover, in a recent review, it is mentioned that PRP should be avoided when the anti-VEGF therapeutic strategy is well planned; nonetheless, in a real-world situation, anti-VEGF treatment adherence is challenging; therefore, PRP is eligible for those eyes with severe DR and for those patients for whom anti-VEGF therapy is not an option due to inadequate adherence [8]. Additionally, as anti-VEGF treatment has no permanent effects, a cost-effective study of each patient must be performed. Also, there is still active research comparing the effectiveness of the two most commonly used anti-VEGF biological drugs: aflibercept and ranibizumab. Similar results are found among them [11,12,13], however, ranibizumab is the more cost-effective treatment in DME in comparison to aflibercept [14]. In contrast, aflibercept is a more effective treatment than ranibizumab when initial visual acuity is moderate to severe [15,16]. Therefore, the aim of the present study is to analyze the effectiveness of aflibercept and ranibizumab in a Mexican population over a span of 5 years in a real-life scenario.

## 2. Material and Methods

### 2.1. Study Design

This is a retrospective real-scenario cross-sectional descriptive study of diabetic patients with intravitreal anti-angiogenic treatment (Ranibizumab [ranib] and/or Aflibercept [aflib]). The study included all medical records that fulfilled all the selected criteria between January 2019 and December 2023. The protocol was evaluated and approved by the Institutional Review Board (CEI-2022-09/05; date of approval: 19 September 2022).

### 2.2. Study Population

*Inclusion criteria:* Medical records were selected from all patients with type-2 diabetes mellitus (T2DM) with a diagnosis of non-proliferative diabetic retinopathy (DR), proliferative diabetic retinopathy (PDR), and diabetic macular edema (DME) who had intravitreal antiangiogenic injections. Also, visual acuity (VA) and/or optical coherence tomography (OCT) before and after antiangiogenic treatment. Routinely, the antiangiogenic treatment schedule is once a month for three months; thereafter, the patient is evaluated, treated, and extended as needed. *Exclusion criteria:* Diagnosis of systemic diseases concomitant to T2DM; inconclusive retinal disease diagnosis; incomplete medical record. *Elimination criteria:* Repeated patient file numbers; patients treated with another intravitreal treatment such as bevacizumab and dexamethasone implants.

### 2.3. Data Collection

To determine the effectiveness of antiangiogenic treatment, VA and OCT parameters were considered. Before antiangiogenic treatment and after 3 months of intravitreal treatment, VA was measured using Snellen fractions, and low visual acuity was assessed by counting fingers (CF), hand movement (HM), color and light perception (LPCP), light perception (LP), and no-light perception (NLP). For the database, the transformation to the *LogMAR* scale was performed using the formula published by Moussa et al., 2021 [17]. OCT scans were obtained using Spectralis OCT (Heidelberg Engineering, Heidelberg, Germany) and Eye Explorer software version 2 (HEYEX 2) (Heidelberg Engineering, Heidelberg, Germany). Central retinal thickness (CRT) (μm) was measured at the center of the fovea; this measure is an average of at least six measurements automatically calculated by the aforementioned device. For VA and OCT, it was measured before (PRE) intravitreal (IV) and after (POST) intravitreal treatment. Data acquisition were reviewed by at least 3 researchers (S.L.-L., O.V.-R. and V.L.-A.).

### 2.4. Statistical Analysis

The results are presented as means and standard deviation (SD), or frequencies. The Kolmogorov-Smirnov test was performed to check for normality. The Wilcoxon test was performed to compare the PRE and POST group treatments. The Mann–Whitney U test was performed to evaluate the differences between diagnoses (DX) and treatments (TX). Data were recorded using Microsoft Excel 365 and added to IBM SPSS Statistics 25 for analysis in addition to PRISM 10.

## 3. Results

### 3.1. Demographics

After reviewing all the medical records between 2019 and 2023, a total of 4323 patients received at least an intravitreal medication, eight hundred and thirty-five subjects had DR and/or PDR and/or DME, and a total of 294 patients fulfilled the inclusion criteria with a total of 588 eyes. The reduced continuity of patient treatment, the absence of data in the registries, and the comorbidities of the patients were the reasons for the exclusion of most of the patients. Women represented 60% (177 patients, 354 eyes) of the sample and men 40% (117 patients, 234 eyes); the mean age was 63.3 ± 9.3 years. Three hundred and eighty-one patients out of the 588 eyes received intravitreal injections; 169 received ranibizumab injections; 99 received aflibercept injections; and 113 received both ranibizumab and aflibercept. From the treated eyes, 195 had VA measurements, while 186 had OCT measurements. The mean number of injections after pre-measurement OCT was 2.91 ± 2.93, and the mean number of injections was for aflibercept 4.06 ± 2.36 and for ranibizumab 2.94 ± 3.05; the mean number of injections when considering patients with both treatments was 2.81 ± 3.16. The data are summarized in Table 1.

### 3.2. Evaluation of Improvement between VA and CRT

**Figure 1:**
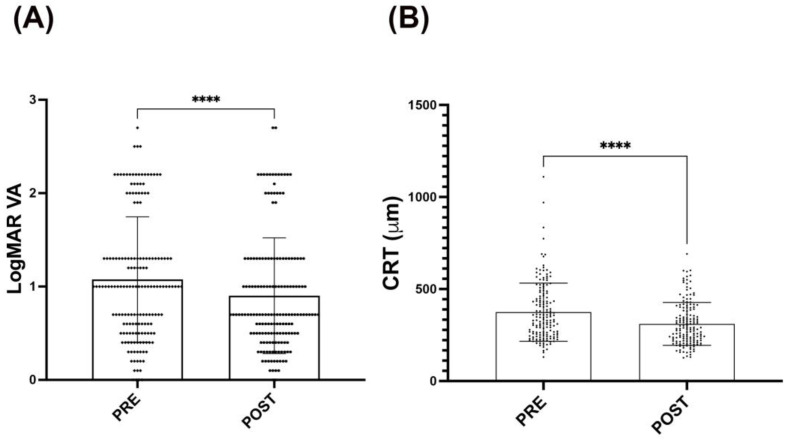
Effect of the antiangiogenic intravitreal injections on VA and CRT measurements. (**A**) Bars graph showing *LogMAR* values before (PRE) and after (POST) antiangiogenic intravitreal injections in all study subjects. (**B**) Bars graph of CRT measurements (μm) before and after antiangiogenic intravitreal injections in all study subjects (**B**). It is observed that there is an improvement in both measurements (VA and CRT) after treatment with antiangiogenic intravitreal injections. A Wilcoxon statistical test was performed. The bars symbolize the mean (±SD). (**** *p* < 0.0001).

In the analysis of patients with anti-angiogenic treatment, it was found that the evaluation by VA and CRT maintains the trend towards improvement after intravitreal antiangiogenic treatment, considering treatments with aflibercept, ranibizumab, or their combination, regardless of the diagnoses (DR, PDR, and DME). Taken together, all pretreatment *LogMAR* values are shown as 1.1 ± 0.66. Interestingly, these values were 0.98 ± 0.60 after intravitreal antiangiogenic, which means a significant (*p* < 0.05) VA improvement. Similarly, behavior presented CRT measurements with OCT; measures in the pretreatment presented 372 ± 152 μm, whilst after treatment, this measurement significantly (*p* < 0.05) decreased around 50 μm (321 ± 171 μm), showing an improvement in the macular thickness (Figure 1).

**Figure 2:**
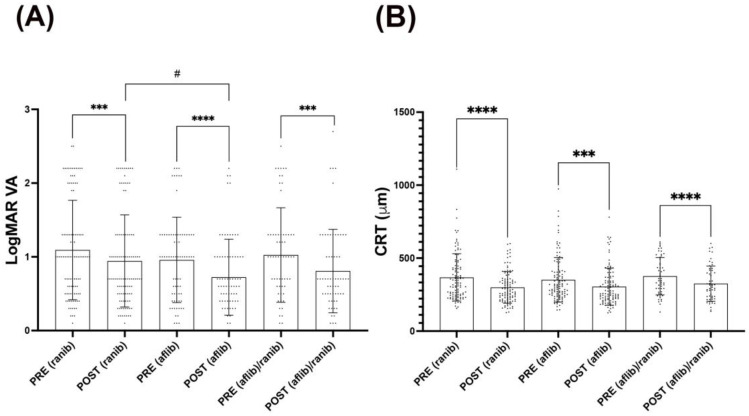
Analysis of the improvement in VA and CRT based on antiangiogenic therapy. Bars graphs representing the distribution of *LogMAR* AV (**A**) and CRT (**B**) values analyzing pre- and post-treatment: ranibizumab [PRE and PRO (ranib)], aflibercept [PRE and PRO (aflib)], ranibizumab/aflibercept [PRE and PRO (ranib/aflib)]. The analysis shows that the treatment of all aflibercept, ranibizumab, and the use of both significantly improved VA and CRT outcomes after antiangiogenic intravitreal injections. Moreover, it was demonstrated to be slightly superior to ranibizumab when VA was taken into account. The bars symbolize the mean (±SD). Wilcoxon statistical test **** *p* < 0.0001; *** *p* < 0.001; U Mann–Whitney test # *p* < 0.05.

To identify whether there were differences among treatments, patients were grouped according to the different antiangiogenic agents to which they were subjected: ranibizumab (ranib), aflibercept (aflib), or a combination of both (ranib/aflib). Antiangiogenic intravitreal injections were effective alone or in combination in both VA and CRT measurements. Although there were statistically significant differences in all the groups when VA was analyzed, aflibercept alone showed the most significant improvement in comparison with ranibizumab alone or ranibizumab in combination with aflibercept; moreover, aflibercept demonstrated to be slightly superior (*p* = 0.028) to ranibizumab when comparisons were made among both post-treatment groups (Figure 2A). Antiangiogenic intravitreal injections were also effective alone and in combination when CRT measurements were analyzed, showing that in the three groups there were statistically significant differences before and after treatments. Interestingly, in this case, ranibizumab intravitreal injection showed the most significant difference compared to the other two groups (aflibercept alone and the combination of both ranibizumab and aflibercept) (Figure 2B). On the other hand, the analysis of the populations according to the treatment used and the number of samples may be affected by factors such as the ophthalmologist’s assessment, the development of the treated condition, and criteria associated with the evolution of the patient, in addition to the number of injections administered. However, in the assumption that the differential population would substantially affect the analyses between treatments, ranibizumab vs. the other conditions would expose the same event in CRT measurements, where it shows no significant differences among group analyses (rani vs. alflib, *p* = 0.79; rani vs. aflib/rani, *p* = 0.24.; aflib vs. aflib/rani, *p* = 0.43).

**Figure 3:**
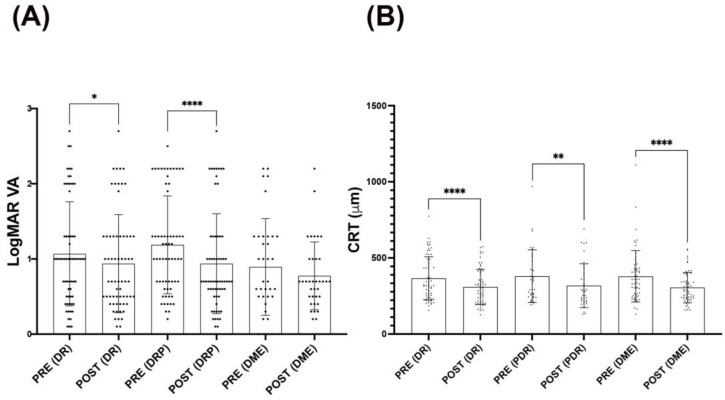
Effect of intravitreal antiangiogenic injections on VA and CRT measurements based on the diabetic retinopathy diagnosis. Bars graphs represent the *LogMAR* (**A**) and CRT (**B**) values based on the retinopathy diagnosis before (PRE) and after (POST) antiangiogenic intravitreal treatments. Diabetic retinopathy (DR), proliferative diabetic retinopathy (PDR), and diabetic macular edema (DME). VA ameliorates in both DR and PDR treated with antiangiogenic intravitreal injections, while this treatment apparently did not modify VA in DME. PDR responded better than DR when VA was measured. Antiangiogenic intravitreal injections ameliorated CRT measurements in all DR, PDR, and EMD retinopathy conditions. The bars symbolize the mean (±SD). Wilcoxon statistical test **** *p* < 0.0001; ** *p* < 0.01; * *p* < 0.05.

Although VA significantly improved after intravitreal antiangiogenic injections (ranibizumab, aflibercept, and ranibizumab/aflibercept) in DR and PDR, antiangiogenic intravitreal treatment did not show any difference in VA in the DME group (Figure 3A). In this context, there is evidence demonstrating the efficacy of anti-VEGF injections on eyes with PDR in improving visual acuity [8]. Moreover, anti-VEGF treatment is believed to have neovascular regression effects in acute PDR subjects [18], and it has also been reported that aflibercept has the capacity to regress the neovascularization process in PDR [19]. Thus, retinal neovascularization regression exerted by anti-VEGF treatments might be a possible mechanism that explains an improvement in DR and PDR without DME.

It is worth mentioning that PDR responded better than DR in terms of statistical values (*p* < 0.0001 vs. *p* < 0.05, respectively) when antiangiogenic intravitreal injections were applied and VA was evaluated. Intravitreal antiangiogenic injections were able to ameliorate CRT measurements in all DR, PDR, and DME conditions, showing that their use was equally effective in DR and DME (Figure 3).

In addition, the behavior of the number of eyes treated with the antiangiogenic agents rani, aflib, and rani/aflib was compared according to the type of condition analyzed, and it was observed that there is a tendency to use rani for DR, PDR, and DME; aflib is more used for DR; and for more complex conditions such as PDR and DME, the use of both antiangiogenic agents (rani/aflib) is chosen (Table 2).

## 4. Discussion

In our study, we analyzed a Mexican population treated with the most commonly used antiangiogenic biologicals, aflibercept and ranibizumab, to treat DR, PDR, and DME. The evaluation of improvement was assessed using the techniques used by ophthalmological personnel to follow up by means of VA and CRT.

Our results indicate that the use of antiangiogenic intravitreal medications helps patients with DR, PDR, and DME by improving visual acuity, a subjective measure that indicates the patient’s perception of vision. Also, the treatment was able to reduce the thickness of the retina by means of OCT, which is a patient-independent procedure and shows results more accurately. It is known that the main effect of antiangiogenic intravitreal treatment is to prevent the progression of the DR but not its reversal, since the reduction in macular thickness does not always correlate with an improvement in VA. It has been reported that even in different stages of the pathology, the relationship between VA and CRT is not entirely similar, as in glaucoma, where macular parameters of CRT were associated with VA in moderate-advanced glaucoma but did not present a difference in early glaucoma [20]. In addition, patients treated with bevacizumab have been evaluated and found that the decrease in central retinal thickness was better in the DME group compared to serous macular detachment but that there was no effect on visual prognosis [21]. Since VA is a clinically significant variable, it bears the most weight when evaluating a patient’s quality of life, and a lack of improvement in VA means that, despite neovascularization and abnormal liquid accumulation leading to visual impairment, the neural damage cannot be reversed through antiangiogenic medications. Although a reduction in macular thickness has been linked clinically, it may not necessarily be a sign of improvement because in diabetic patients, it has been associated with diabetic peripheral neuropathy [22].

Successful treatment of DR and DME will come with a better understanding of the underlying pathogenesis. Many articles in the literature attempt to explain other causes of the pathogenesis and include immunological, neural, and genetic factors, as well as metabolic syndrome, as multifactorial culprits for the lack of success of anti-VEGF therapies and the overall microvascular complications of diabetes [7,23].

Antiangiogenic medications have been studied at length, and observations include a lack of progression of DR and less presentation of DME. Overall, ranibizumab has been shown to be a safe and good option for the treatment of advanced DR stages, high-risk DR, and DME; nevertheless, in our study, a lack of response to ranibizumab prompted a change to aflibercept. By analyzing which anti-VEGF medication was better, we found that in a meta-analysis by Virgili E., and colleagues., in which they analyzed three anti-VEGF medications and PRP, they established that aflibercept and ranibizumab had better outcomes than both PRP alone and bevacizumab, and between them, aflibercept had better outcomes [24]. Similarly, the CLARITY study showed that PDR eyes treated with aflibercept had better VA outcomes at 1 year compared with eyes treated with PRP, and that aflibercept was superior to PRP [25].

In our study, when assessing improvement, we found that it responds positively regardless of treatment, but when comparing the treatment groups, it was found that aflibercept has a greater improvement compared to ranibizumab or the use of both in the measurement of VA. Although more doses of aflibercept were administered on average, it is also important to consider that the population that is hardly able to pay for the treatment needs to focus on the treatment they can afford. In most cases, the most economical method is chosen. Many of the patients had undergone PRP treatments prior to treatment. The addition of antiangiogenic intravitreal use to PRP treatment has been reported to significantly increase its improvement, according to several meta-analyses [26,27]. However, there have been reported cases where the ophthalmologist decides to switch from PRP to use anti-VEGF mid-treatment. Studies have shown that delayed cross-treatment is not as promising as the results of rapid anti-VEGF treatment [28]. That is why studies are needed to support individual or combined use to avoid neovascularization, in addition to having a follow-up of patients, since in several cases the treatment is interrupted, changed from one to another due to a change in costs, or simply abandoned.

Most of the observed improvement was related only to the thickness of the macula, where the treatment has its main effect, but the VA results consider the patient’s perception, which often differs because it depends on factors that the ophthalmologist does not control, such as day, time, and emotional state, among many others, which could sometimes cause a false positive, overestimating the VA measurement. Therefore, in some cases, the data are contradictory between the VA and the CRT measurement. In addition, it supports the idea that the progression and sequelae of DR depend not only on angiogenic status but also on the multifactorial nature of the disease, such as damage to the nervous system, the immune system, and the changes in the extracellular matrix caused by diabetes per se [6]. Even genetic factors, which are generally standardized, do not take into account population factors and the different risk factors implicit in the population [29]. Furthermore, it has been reported that eyes with PDR treated with anti-VEGF alone may experience marked disease progression with potentially serious visual consequences if treatment is discontinued for uncontrolled or conscious reasons [30]. However, an individualized treatment that depends on the patient’s adherence, the economic burden, and the distances to reach the healthcare center are determining factors in obtaining successful results.

The major limitation of our study is that it is based on a real-world scenario. The three-dose scheme is dependent on the economic status of the patients or if they have an insurance coverage policy. These factors determine the adherence to the treatment; therefore, to our observation, this situation is the main reason patients do not follow the suggested intravitreal treatment in the present study. In this context, we are aware that in many countries, intravitreal treatment success is really dependent on insurance support.

One of the main problems lies in the fact that treatments require greater durability and efficacy. The treatment of DME and DR requires modifying the paradigms that are established in these conditions and proposing new pathways and molecules involved in their pathogenesis. Even new anti-VEGF therapies have been implemented, such as faricimab, which has been applied to patients with DME and has a favorable prognosis, but even in this case, the regimen is based on 12 weeks [31]. In this context, it is worth mentioning that faricimab is now registered in Mexico, and more studies need to be performed in our population to determine its efficacy. In the assessment of new drugs, they should be evaluated in an appropriate form for specific populations, since in different reports it has been suggested that conditions like DR have a racial factor. In this context, it has been described that the prevalence of DR in patients with T2DM is 46% higher in non-Hispanic blacks and 84% higher in Mexican Americans than in Caucasians [32]. The population ethnicity is important considering the great variability observed in different features such as incidence, progression, heritability, pathogenesis, and response to treatment [33].

The lack of improvement in a large percentage of patients is what prompts the search for other explanations for the pathogenesis of DR. Thus far, we know that patients with DR also have prior neural damage, sometimes to the appearance of DME or neovascularization, due to the nature of the pathogenesis of diabetes, which in our country is commonly found combined with systemic hypertension, which was found to worsen the damage to visual structures, and which, combined with the increased glucose levels, affects not just the cells but the extracellular matrix, making injuries worse, recovery slower or impossible, and dysregulation of the immune system. This is also because nerve damage is one of the main complications caused by diabetes [6,34,35,36].

The importance of this study is to understand the current state of anti-angiogenic treatments at an ophthalmologic reference center in Mexico. Patients with a diagnosis of DR, PDR, or DME, which are complications of T2DM, a condition with a high prevalence in Mexico (18%), were evaluated, in addition to encouraging other reference centers to evaluate the efficacy of anti-angiogenic agents in the Mexican population. Although the Mexican population has been evaluated in international or Latin American studies [37], most categorize them as Hispanic, grouping them with other ethnicities such as Brazil, which has a high incidence of T1DM, which makes it difficult to compare the real effect of anti-angiogenics. Hence, it is important to start promoting the effects of these treatments on the Mexican population.

Finally, we are convinced that visual acuity and central retinal thickness measurements are indirect indexes to evaluate the antiangiogenic effect of the intravitreal drugs. Although we have OCT-angiography (OCT-A) technology, this technique is not routinely achieved due to its high cost; therefore, OCT-A measurements were not included in this study.

## 5. Conclusions

This study, along with several others, predominantly demonstrates the importance of early diagnosis and control of diabetes because, although the treatments exist and are somewhat successful, they do not reverse the damage done to visual structures provoked by the disease. Despite the macula reducing its thickness, VA does not improve in at least half of the patients. This study is only a small view of the large problem facing a large portion of the diabetic population. To understand the real impact of this disease in countries such as Mexico, where diabetes presents a high prevalence and complications due to comorbidities are common, the lack of specific treatment for the Mexican population, the reduced integration between health institutions to maintain a consensus on treatment, and the lack of a central database with patients’ medical records are hindrances to establishing the real impact of diseases like DR on the population and health services. The creation of a database focusing on diabetic patients would help lawmakers and healthcare institutions establish prevention programs and allocate resources to treat patients before they present the worst complications from diabetes.

## Figures and Tables

**Table 1:** Demographic characteristics of the involved subjects in the study.

	*N* = 294 (588 eyes)	
	*n*	%
***Gender***		
Female	177 (354 eyes)	60%
Male	117 (234 eyes)	40%
	Mean ± SD	**CI**
***Age***	63.39 ± 9.3	62.32–64.48
Female	64.32 ± 9.4	62.98–65.78
Male	61.91 ± 9.1	60.25–63.58
***Diagnoses***		**%**
DR	242	41.2%
DME	155	25.6%
PDR	191	32.4%
***Treatment***		
IV (Total)	381	64.7%
Aflibercept	99	25.9%
Ranibizumab	169	44.4%
Aflib/ranib	113	29.7%
***Measurements***		
VA	195	51.1%
OCT	186	48.9%
***Follow up OCT***		
Mean No. Injection	2.91 ± 2.93	
Mode No. Injection	3	
***Number of Injections***		
Ranibizumab	2.94 ± 3.05	
Aflibercept	4.06 ± 2.36	
Aflib/ranib	2.81 ± 3.16	

DR: diabetic retinopathy; DME: diabetic macular edema; PDR: proliferative diabetic retinopathy; VA: visual acuity; OCT: optical coherence tomography.

**Table 2:** Number of eyes with different pathologies treated with antiangiogenic agents.

	DR	PDR	DME	Total
Rani	70	55	44	169
Aflib	43	27	29	99
Rani/aflib	37	36	40	113
Total	150	118	113	381

## Data Availability

The data presented in this study are available on request from the corresponding author (the dataset contains private information protected by law).
